# Association between the MMP-1-1607 1G/2G Polymorphism and Osteoarthritis Risk: A Systematic Review and Meta-Analysis

**DOI:** 10.1155/2020/5190587

**Published:** 2020-05-20

**Authors:** Jiankun Liu, Guangye Wang, Zhan Peng

**Affiliations:** ^1^Department of Spine Surgery, Tianjin Hospital, No. 406, Jie Fang Nan Road, Hexi District, Tianjin 300211, China; ^2^Department of Spinal Surgery, The Second Affiliated Hospital of Shenzhen University, Shenzhen Baoan District People's Hospital, Shenzhen 518101, China

## Abstract

**Background:**

Osteoarthritis (OA) is a common disease characterized by articular cartilage degeneration and secondary hyperosteogenesis. Genetic factors are associated with the occurrence of OA. While several studies have shown that the matrix metalloproteinase-1- (MMP-1-) 1607 1G/2G (rs1799750) polymorphism may be related to the occurrence and development of OA, there is inconsistency in the literature. To better estimate the relationship between the MMP-1 gene polymorphism and OA, a comprehensive meta-analysis of relevant literature was carried out.

**Results:**

In total, seven studies comprising 1245 OA patients and 1230 controls were included in this meta-analysis. The combined results revealed no significant association between the MMP-1-1607 1G/2G polymorphism and risk of OA in the five genetic models. However, after Bonferroni correction, the results of subgroup analysis revealed a significant correlation between the MMP-1-1607 1G/2G polymorphism and OA susceptibility in the temporomandibular joint (TMJ) OA subgroup (allelic: 2G vs. 1G: OR = 1.575, 95%CI = 1.259–1.972, *P* < 0.01; recessive: 2G2G vs. 1G1G+1G2G: OR = 2.411, 95%CI = 1.658–3.504, *P* < 0.01; and homozygote: 2G2G vs. 1G1G: OR = 2.313, 95%CI = 1.341, 3.991, *P* = 0.003), the younger subgroup (aged less than 60 years; allelic: 2G vs. 1G: OR = 1.635, 95%CI = 1.354, 1.974, *P* < 0.01; dominant: 2G1G+2G2G vs. 1G1G: OR = 1.622, 95%CI = 1.158, 2.271, *P* = 0.005; recessive: 2G2G vs. 1G1G+1G2G: OR = 2.209, 95%CI = 1.718, 2.840, *P* < 0.01; and homozygote: 2G2G vs. 1G1G: OR = 2.578, 95%CI = 1.798, 3.696, *P* < 0.01), the larger subgroup (*N* > 300), and the hospital-based case-control study (HCC) subgroup. The sensitivity analysis suggested that the results of the meta-analysis were stable and reliable. Begg's funnel plot and Egger's test indicated that there was no publication bias in this study.

**Conclusion:**

Our meta-analysis indicated that although the MMP-1-1607 1G/2G polymorphism was not significantly associated with OA susceptibility among the whole sample, it played a key role in the etiology and development of TMJ OA and OA in people aged less than 60 years.

## 1. Background

Osteoarthritis (OA) is a cartilage degenerative disease that is more common among middle- and older-aged people. The main pathological manifestations include degeneration of articular cartilage, formation of osteophytes, and subchondral bone sclerosis [1]. Clinical manifestations include slow-developing joint pain, stiffness, joint swelling, and joint deformities. The incidence of OA worldwide remains high. The disease significantly limits a patient's life, causing unbearable pain and even disability, and imposes an immense burden on society and the families of patients [2]. The etiology and pathogenesis of OA are not completely clear and appear to involve complex interactions between genetic and environmental factors. Studies have shown that, in addition to factors such as age, sex, and weight, many cytokines are also implicated in the pathological process of OA [3].

The role of matrix metalloproteinase-1- (MMP-1-) mediated destruction of articular cartilage in the pathogenesis of OA has attracted widespread attention. MMP-1, also known as collagenase 1, is the most widely expressed proteolytic enzyme in the MMP family. MMP-1 is primarily produced by interstitial cells, epithelial cells, and endothelial cells. Articular cartilage is composed of chondrocytes and extracellular matrix (ECM), and MMP-1 can degrade collagens I, II, and III in ECM. Thus, when MMP-1 is overexpressed in chondrocytes, there is an increase in the degradation of cartilage collagen and proteoglycan, resulting in pathological cartilage damage; this underlies the development of OA [4, 5]. Expression of MMP-1 in OA chondrocytes is higher than that in normal chondrocytes [6]. Taken together, these findings suggest that MMP-1 is closely related to the pathogenesis and pathological process of OA.

Single nucleotide polymorphisms (SNPs) are polymorphisms of DNA sequences caused by single nucleotide variation at the gene level [7]. SNPs located in the region of the MMP-1 promoter can affect the transcription level of genes, thereby increasing or decreasing the expression of genes and playing an important role in the pathogenesis of related diseases [8]. The -1607 SNP (1G/2G, rs1799750) in the MMP-1 promoter region was the first and most widely researched SNP in relation to OA. This SNP is caused by guanine insertion/deletion and affects the expression of MMP-1 and the degradation of matrix [8].

Barlas et al. first studied the 1G/2G site of the MMP-1-1607 gene in Turkish patients with OA. It was reported that the MMP-1 gene polymorphism may be associated with susceptibility to OA; in particular, the 1G allele and 1G gene model were associated with susceptibility to OA. Subsequently, many researchers attempted to replicate this finding; however, the results were inconsistent [9–15]. Given the limited sample sizes and racial differences among the samples in the literature, it is difficult to draw generalized conclusions. Therefore, we performed a meta-analysis of all relevant research data to quantitatively assess the potential association between OA and the MMP-1-1607 1G/2G polymorphism.

## 2. Materials and Methods

### 2.1. Literature Search

Manual searches of the PubMed, Embase, China Knowledge Network, and Wanfang databases were performed. The following keywords were used to identify relevant literature: “MMP-1 or matrix metalloproteinase-1,” “polymorphism or variation,” “rs1799750 or MMP-1 1607,” “susceptibility or risk,” and “osteoarthritis or joint degeneration.” In addition, we also carried out literature tracing; that is, we used reference lists and citations in the relevant retrieved literature to trace and identify further relevant literature that was not identified in the database searches. There was no language restriction on the literature search. The last retrieval date for relevant studies was July 2019. Two researchers used the same retrieval method to search the databases independently. When there was a disagreement, they negotiated a solution.

### 2.2. Inclusion and Exclusion Criteria

The inclusion criteria included (1) original case-control studies examining the relationship between the MMP-1-1607 1G/2G (rs1799750) polymorphism and susceptibility to OA, (2) all cases with a diagnosis of OA and a control group composed of healthy individuals or individuals without a family history of OA, (3) genotype distribution in the control group consistent with the Hardy-Weinberg equilibrium (HWE), and (4) enough genotypic data for the case group and control group to calculate odds ratios (ORs) and 95% confidence intervals (CIs). The exclusion criteria included (1) duplicate studies, (2) non-case-control studies, and (3) failure to provide detailed genotypic frequency data. In addition, we excluded unpublished reports, abstracts, reviews, and editorial comments. Two researchers independently judged the identified research according to the inclusion and exclusion criteria. Disputes were settled via discussion among the researchers.

### 2.3. Data Extraction

The information extracted from each included study is described in Tables 1 and 2; this information included the first author, publication year, country, race, OA site, sample, study design, detection method of gene, sample size in the case and control groups, frequency of genotypes and alleles in the case group and the control group, and the results of the HWE test (Pearson's chi-square test was used to detect whether the genotype distribution of the control group was in accordance with the HWE; *P* < 0.05 was a significant deviation from the HWE). The data extraction was carried out independently by two researchers using a standardized form; disputes were resolved via discussion.

### 2.4. Evaluation of Literature Quality

Quality evaluation of the included literature was carried out independently by the two researchers. Since the literature included in the present study comprised observational case-control studies, the Newcastle-Ottawa Scale (NOS) was used to evaluate the quality of the included literature. The maximum score of the NOS is nine. Studies scoring greater than five points can be included in a meta-analysis. We considered studies with scores greater than or equal to seven to be of high quality [16].

### 2.5. Statistical Analysis

Pooled ORs and 95% CIs were used to evaluate the relationship between MMP-1-1607 1G/2G (rs1799750) and OA susceptibility. We evaluated the following five genetic models: allele genetic model (2G vs. 1G), homozygote genetic model (2G2G vs. 1G1G), heterozygote genetic model (2G1G vs. 1G1G), dominant genetic model (2G1G+2G2G vs. 1G1G), and recessive genetic model (2G2G vs. 1G1G+1G2G).

The *Q* test based on the chi-square test was used to evaluate heterogeneity among the included studies [17]. When *P* < 0.1, statistically significant heterogeneity was assumed, and a random effects model (DerSimonian-Laird) [18] was used for the pooled analysis. Otherwise, a fixed effects model (Mantel-Haenszel method) was used for the pooled analysis [19]. If *P* < 0.05, the pooled OR value was considered meaningful. To maximize the avoidance of possible false positive results in the subgroup analysis, we also performed strict Bonferroni correction for *P* values less than 0.05 [20].

When significant heterogeneity was present, the possible sources of heterogeneity were identified by metaregression analysis. For these analyses, logistic regression was performed with the OR as the dependent variable and the factors that might influence heterogeneity, such as ethnicity, location of OA, study design, patient surgery, sample size, average age, and study quality score, as the independent variables. In these analyses, *P* < 0.05 indicated that the corresponding independent variable was the source of heterogeneity [21].

We also conducted subgroup analysis based on ethnicity, location of OA, study design, patient surgery, sample size, average age, and study quality to evaluate the possible effects of different subgroups on the results of the study. In addition, we performed sensitivity analysis to investigate the effect of each single study on the pooled effect to explore whether a single study was the source of heterogeneity. We used Begg's funnel plot and Egger's test to identify possible publication bias in our meta-analysis. A nonsymmetrical funnel plot or *P* < 0.05 indicated that there was significant publication bias. All statistical analyses were carried out using STATA 14.1 software (Stata Corporation, College Station, TX, USA).

## 3. Results

### 3.1. Study Selection and Characteristics of Included Studies

**Figure 1:**
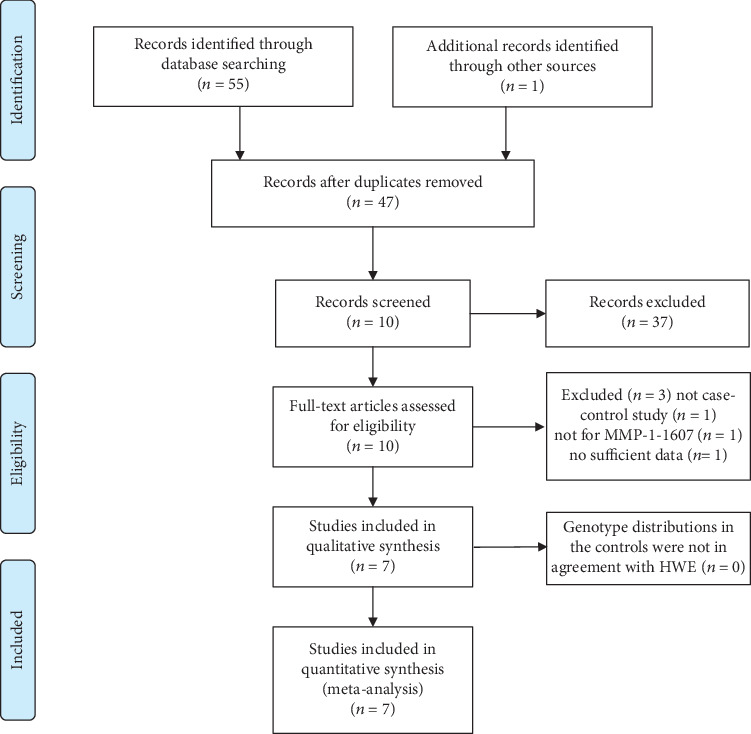
Flow diagram of the study selection process.

Fifty-five potentially relevant articles were retrieved. After initial exclusion of eight duplicate documents, 37 articles with unmatched titles and abstracts were excluded. Finally, the full texts of the remaining 10 articles were carefully read. One study did not provide enough genotypic data to calculate the OR and 95% CI [22], one was not a case-control study [23], and one was not a study of the MMP-1 1G/2G gene polymorphism [24]; thus, these studies were excluded. Finally, seven articles remained for inclusion in the meta-analysis. Data were extracted from these papers, and the meta-analysis was carried out [9–15]. The seven studies comprised 1245 patients with OA and 1230 controls. The process for searching and selecting relevant articles is shown in Figure 1. Most of the included studies were of knee OA; only two studies contained some cases of temporomandibular joint (TMJ) OA [12, 14]. Quality assessment of studies included in the meta-analysis showed that the NOS of all literatures was greater than 5. The basic characteristics of each study, the quality evaluations, and the genotypes of the included patients are shown in Tables 1 and 2 and Supplementary Table 1.

### 3.2. Meta-Analysis of the Relationship between the MMP-1-1607 1G/2G Polymorphism and Risk of OA

In the total sample, the MMP-1 1G/2G polymorphism was not found to be significantly associated with risk of OA in the five genetic models (OR(95% CI): allelic: 1.232 (0.889, 1.707), *P* = 0.21; recessive: 1.478 (0.925, 2.6), *P* = 0.102; dominant: 1.203 (0.801, 1.807), *P* = 0.373; heterozygote: 1.512 (0.828, 2.761), *P* = 0.179; and homozygote: 1.088 (0.774, 1.529), *P* = 0.627).

**Figure 2:**
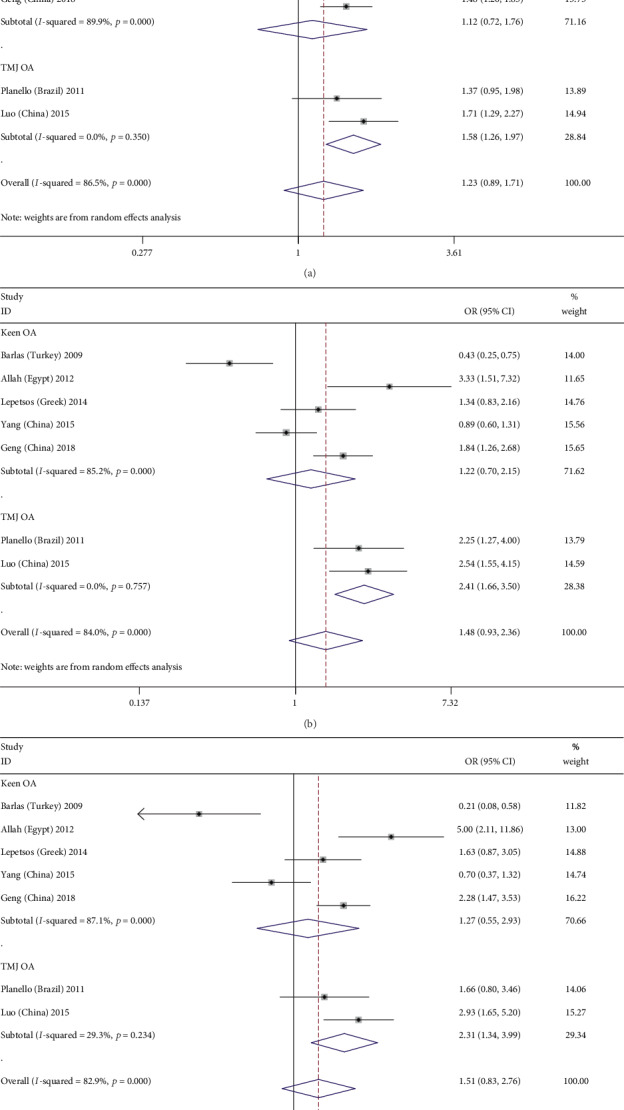
Forest plots of MMP-1-1607 1G/2G polymorphism and osteoarthritis risk in three genetic models stratified by the osteoarthritis site. (a) Allele genetic model: 2G versus 1G; (b) recessive genetic model: 2G2G versus 1G1G+1G2G; (c) homozygote genetic model: 2G2G versus 1G1G.

**Figure 3:**
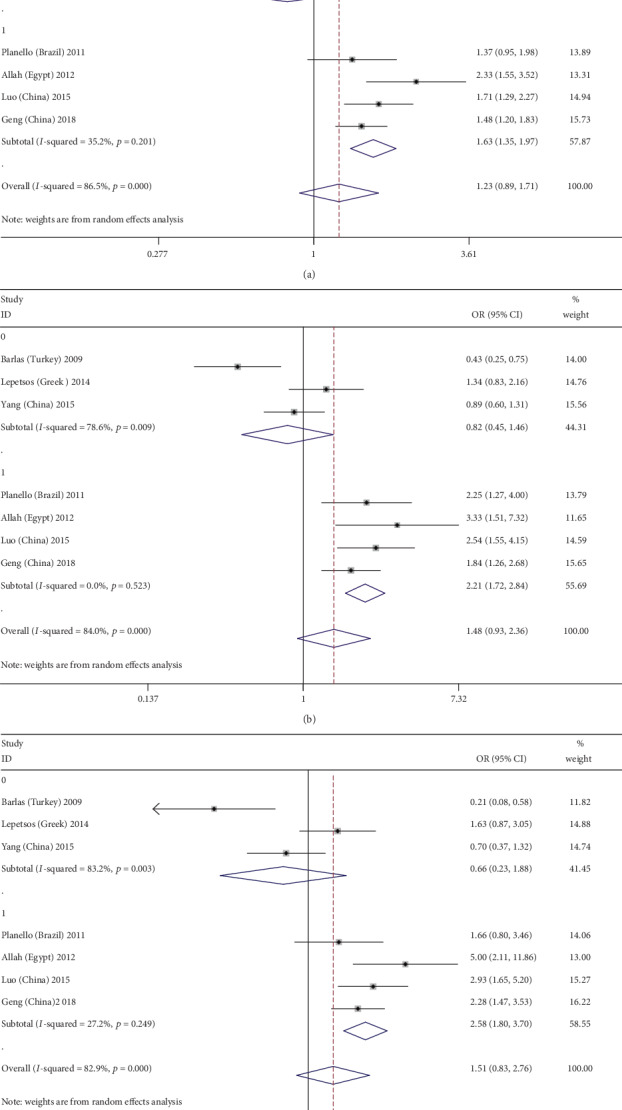
Forest plots of MMP-1-1607 1G/2G polymorphism and osteoarthritis risk in three genetic models stratified by mean age (0: over 60 years old; 1: under 60 years old). (a) Allele genetic model: 2G versus 1G; (b) recessive genetic model: 2G2G versus 1G1G+1G2G; (c) homozygote genetic model: 2G2G versus 1G1G.

The results of the subgroup analyses showed that there was a significant correlation between the MMP-1-1607 1G/2G polymorphism and OA susceptibility in the temporomandibular joint (TMJ) OA subgroup after Bonferroni correction (when *P* < 0.025, the pooled OR value was considered meaningful) (allelic: 2G vs. 1G: OR = 1.575, 95%CI = 1.259–1.972, *P* < 0.01; recessive: 2G2G vs. 1G1G+1G2G, OR = 2.411, 95%CI = 1.658, 3.504, *P* < 0.01; and homozygote: 2G2G vs. 1G1G: OR = 2.313, 95%CI = 1.341, 3.991, *P* = 0.003) (Figures 2(a)–2(c)), the younger subgroup (aged less than 60 years; allelic: 2G vs. 1G: OR = 1.635, 95%CI = 1.354, 1.974, *P* < 0.01; dominant: 2G1G+2G2G vs. 1G1G: OR = 1.622, 95%CI = 1.158, 2.271, *P* = 0.005; recessive: 2G2G vs. 1G1G+1G2G: OR = 2.209, 95%CI = 1.718, 2.840, *P* < 0.01; and homozygote: 2G2G vs. 1G1G: OR = 2.578, 95%CI = 1.798, 3.696, *P* < 0.01) (Figures 3(a)–3(c)), the larger subgroup (*N* > 300; recessive: 2G2G vs. 1G1G+1G2G: OR = 1.588, 95%CI = 0.872, 2.894, *P* = 0.003), and the hospital-based case-control study (HCC) subgroup (recessive: 2G2G vs. 1G1G+1G2G: OR = 1.713, 95%CI = 1.054, 2.783, *P* = 0.03). The detailed results are shown in Table 3.

### 3.3. Heterogeneity and Sensitivity Analysis

Heterogeneity was found in all five genetic models; thus, random effects models were used for all five analyses. We performed metaregression analysis to identify the source of heterogeneity. Unfortunately, common variables such as ethnicity, location of OA, method, study design, patient surgery, sample, total size, mean age, and study quality were not found to be the sources of heterogeneity. Detailed results are shown in Supplementary Table 2.

Nonetheless, we still carried out subgroup analyses according to ethnicity (Caucasian, Asian), location of OA (keen OA, TMJ OA), total sample size (*n* < 300, *n* ≥ 300), study design (HCC or population-based case-control study (PCC)), and age (<60 years old, ≥60 years old) as we considered these analyses to be valuable. Subgroup analyses showed that the heterogeneity in all contrast models was significantly decreased in the TMJ subgroup and the older age subgroup. Therefore, the location of OA and the age of the patients are possible sources of heterogeneity. Detailed results are shown in Table 3.

**Figure 4:**
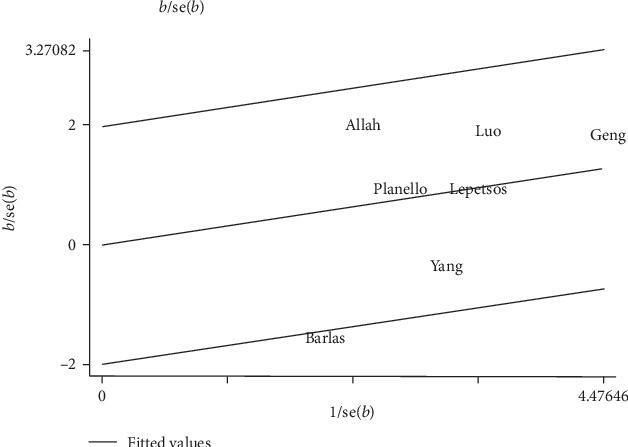
Galbraith plots of MMP-1-1607 1G/2G polymorphism and osteoarthritis risk in the homozygote genetic model: 2G2G versus 1G1G.

Galbraith plot analysis indicated that the study by Barlas et al. was an outlier and the main contributor to the heterogeneity in all contrast models (Figure 4). However, the heterogeneity remained the same after excluding this outlier study (2G vs. 1G *I*^2^ = 73.30%, *P*_heterogeneity_ = 0.002; 2G1G+2G2G vs. 1G1G *I*^2^ = 58.60%, *P*_heterogeneity_ = 0.034; 2G2G vs. 1G1G+1G2G *I*^2^ = 72.10%, *P*_heterogeneity_ = 0.003; 2G2G vs. 1G1G *I*^2^ = 71.10%, *P*_heterogeneity_ = 0.004; and 2G1G vs. 1G1G *I*^2^ = 48.00%, *P*_heterogeneity_ = 0.087).

**Figure 5:**
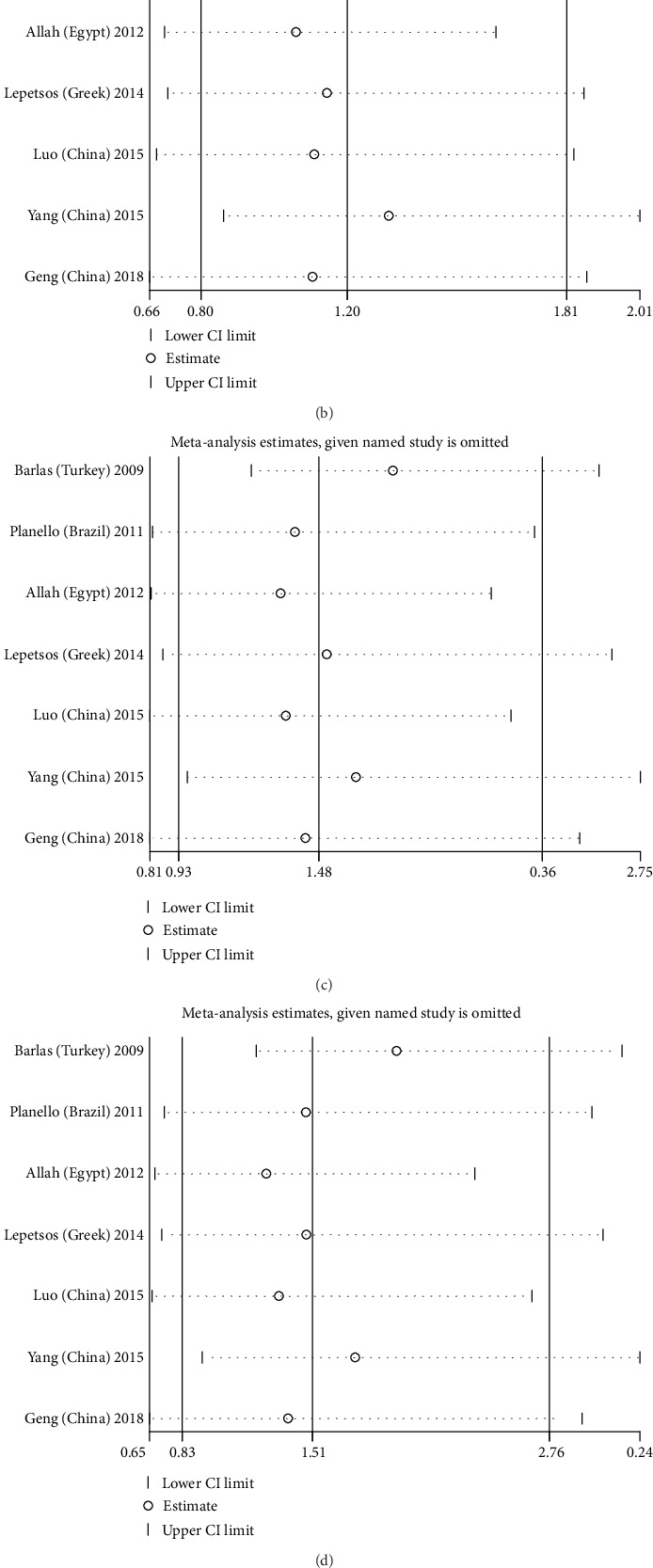
Sensitivity analysis in five genetic models: (a) Allele genetic model: 2G vs. 1G; (b) dominant genetic model: 2G1G+2G2G vs. 1G1G; (c) recessive genetic model: 2G2G vs. 1G1G+1G2G; (d) homozygote genetic model: 2G2G vs. 1G1G; (e) heterozygote genetic model: 2G1G vs. 1G1G.

Next, we conducted sensitivity analysis to explore the effects of each study on heterogeneity and pooled effects. The results showed that when any one study was eliminated and the remaining studies were included in a meta-analysis, there was no significant change in the pooled effect. This suggests that the results of the meta-analysis are stable and reliable (Figures 5(a)–5(e)); however, there was still significant heterogeneity among the studies.

### 3.4. Publication Bias Analysis

**Figure 6:**
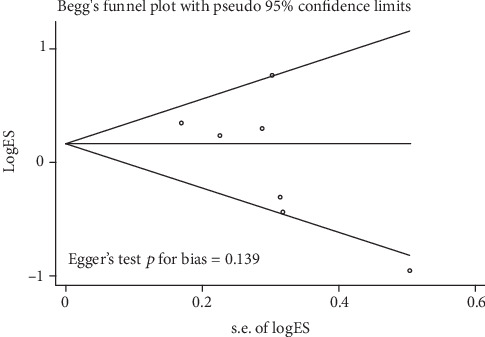
Funnel plot analysis and Egger's test to detect publication bias for contrast 2G1G+2G2G versus 1G1G of MMP-1-1607 1G/2G polymorphism in overall analysis. Each point represents a separate study for the indicated association.

Begg's funnel plot and Egger's test were used to evaluate the presence of publication bias. Moreover, all *P* values for Egger's test and Begg's test for the five genetic models were greater than 0.05, indicating that there was no significant publication bias in the present study. Taking the allele contrast model (2G vs. 1G) as an example, we analyzed the funnel plots and found no apparent asymmetry (Figure 6).

## 4. Discussion

OA is a chronic joint disease with many etiological factors. The progressive destruction of articular cartilage underlies OA [10]. Articular cartilage consists of chondrocytes and ECM. MMP-1 can degrade collagen fibers in the ECM of articular cartilage and plays an important role in the pathogenesis and course of OA [4]. The insertion and deletion of guanine at the -1607 position were observed in the promoter of the human MMP-1 gene. Rutter et al. first proposed that a 2G promoter sequence can form a sequence of erythrocyte-specific (ETS) family-binding sites of transcriptional factors with adjacent A, that is, 5'-GGAA-3. Under the interaction of this sequence with adjacent AP-1 and PEA-3 elements, promoter activity and transcription level of the MMP-1 gene are significantly increased [8, 25], even by as much as 2-10 times [26]. Given the observed degradation of collagen fibers in articular cartilage by MMP-1 and the functional SNPs of the MMP-1 promoter sequence, the relationship between the MMP-1-1607 1G/2G polymorphism and OA has been extensively studied; however, the findings of these studies are inconsistent [9–15].

The inconsistency in findings has been discussed in two published meta-analyses; however, the conclusions of these meta-analyses were inconsistent. In 2016, Xu et al. [27] found that the -1607 1G>2G polymorphism may increase the risk of OA. Subsequently, Xu et al. [28] reported that the -1607 1G>2G polymorphism was not associated with OA susceptibility. Yet, neither of these meta-analyses included all relevant studies. A study of knee OA published by Barlas et al. in 2009 [10] was not included in the Xu et al. study [27], while a study on TMJ OA published in 2011 [12] was not included in the Xu et al. meta-analysis [27]. In addition, since the last published meta-analysis, a large study has been published that links the -1607 1G>2G SNP with OA risk; this study may significantly impact on the results of a meta-analysis. Therefore, we performed a meta-analysis that brought together all eligible studies published to date to more accurately estimate the relationship between the -1607 1G/2G SNP and OA.

A total of seven studies were included in this meta-analysis, comprising 1245 patients with OA and 1230 controls. The results indicated that the MMP-1-1607 1G/2G gene polymorphism was not associated with OA susceptibility in all five genetic models, and there was no significant increase in the risk of OA in those with the 2G gene compared with those without the 2G gene. These findings are consistent with Yang et al. [9] who found no significant association between the MMP-1-1607 1G/2G gene polymorphism and OA susceptibility in an Asian population. However, Abd-Allah [15] studied the MMP-1-1607 1G/2G gene polymorphism in patients with OA and healthy controls and reported that the frequency of haploid 2G in OA was twice as high as that in healthy controls. We believe that sample size, genotyping technique, and selection criteria may be important factors causing the variation in findings in the literature. Compared with the above study, our meta-analysis has a large sample size and greater statistical ability to provide a more accurate conclusion.

In our analysis, we observed heterogeneity among the included studies. Therefore, we carried out metaregression analysis to identify the source of heterogeneity. Unfortunately, common variables such as ethnicity, location of OA, study design, patient surgery, sample size, average age, and study quality were not found to be important sources of heterogeneity. Nonetheless, we still carried out subgroup analysis according to ethnicity, location of OA, total sample size, design (HCC or PCC), and age as these analyses are valuable. We found significant differences in the TMJ OA subgroup analysis in the allelic gene model, recessive gene model, and homozygous gene model; this may be due to differences in the pathogenesis of OA in different regions of the body, suggesting that MMP-1 plays a more important role in the pathogenesis of TMJ OA. Similarly, the younger age subgroup (less than 60 years old) showed that the MMP-1-1607 1G/2G gene polymorphism was associated with OA susceptibility in the younger age group in the allelic gene model, dominant gene model, recessive gene model, and homozygous gene model. Although the reasons for this finding are unclear, we suspect that OA patients of different ages may have different underlying pathogenesis. For young patients, the role of genetic factors in the pathogenesis of OA may be more important. As patients get older, they may be affected by an accumulation of environmental and lifestyle factors, and the effects of genes on OA susceptibility may become weaker.

Our meta-analysis has several advantages. First, to the best of our knowledge, this is the most comprehensive meta-analysis of the relationship between the MMP-1-1607 1G/2G gene polymorphism and OA risk. All relevant data were included in the meta-analysis and subgroup analysis, sensitivity analysis, and metaregression analysis which were carried out to provide more comprehensive and reliable results. Secondly, based on the quality evaluation, all included studies are of high quality. Finally, Begg's funnel plot and Egger's test confirmed that there was no publication bias evident.

There are several limitations in our meta-analysis that should be noted. First, the number of studies included in this meta-analysis is small, especially with respect to the reliability of subgroup analysis; thus, it may not be possible to fully evaluate the true association between the MMP-1-1607 1G/2G gene polymorphism and risk of OA. Second, due to the inability to obtain the original data included in the study, it is impossible to adjust the analyses for other variables such as sex and body mass index (BMI), which may affect the accuracy of the results. Third, in some genetic models, we found significant intergroup heterogeneity. Although we used metaregression analysis, subgroup analysis, and sensitivity analysis to identify the source of heterogeneity and minimize its impact, heterogeneity is still an inevitable problem affecting the accuracy of the results.

In summary, this meta-analysis assessed all relevant published data on the association between the MMP-1-1607 1G/2G polymorphism and OA risk. Our meta-analysis showed that although the MMP-1-1607 1G/2G polymorphism was not significantly associated with OA susceptibility in the whole sample, it played a key role in the etiology and development of TMJ OA and OA in people aged under 60 years.

## Figures and Tables

**Table 1:** Characteristics of the eligible studies included in the meta-analysis.

Author	Year	Country	Ethnicity	OA site	Sample	Genotyping method	Surgery	Design	Sample size			Gender (M/F)		Mean age		NOS
									Total size	Case	Control	Case	Control	Case	Control	
Barlas	2009	Turkey	Caucasian	Keen OA	Blood	PCR-RFLP	No	HCC	241	157	84	36/121	25/59	41–86; 61.7 ± 9.2	41–75; 52.36.9	6
Planello	2011	Brazil	Caucasian	TMJ OA	Buccal cell	PCR-RLFP	No	PCC	232	115	117	14/101	15/102	42.82 ± 14.96	38.0 ± 14.17	7
Abd-Allah	2012	Egypt	Caucasian	Keen OA	Blood	PCR-RLFP	No	HCC	200	100	100	40/60	45/55	36–66; 51.4 ± 8.1	33–67; 54.2 ± 9.4	6
Lepetsos	2014	Greek	Caucasian	Keen OA	Blood	PCR-RLFP	Yes	HCC	294	155	139	33/122	42/97	73.1 ± 7.2	73.8 ± 11.5	8
Luo	2015	China	Asian	TMJ	Buccal cell	HRMA	Yes	PCC	391	206	185	NA	26/159	16–75; 37.22 ± 15.555	16–71; 33.497 ± 10.830	6
Yang	2015	China	Asian	Keen OA	Blood	PCR-RFLP	Yes	PCC	414	207	207	53/154	53/154	70.08 ± 7.41	71.03 ± 7.76	8
Geng	2018	China	Asian	Keen OA	Blood	PCR-RFLP	No	PCC	707	306	401	164/142	199/202	51.42 ± 6.69	51.64 ± 7.02	7

OA: osteoarthritis; TMJ: temporomandibular joint; PCR-RFLP: polymerase chain reaction-restriction fragment length polymorphism; HRMA: high-resolution melting assay; HCC: hospital-based case-control study; PCC: population-based case-control study; NOS: Newcastle-Ottawa Scale.

**Table 2:** Genotype and allele distributions of MMP-1-1607 1G/2G (rs1799750) polymorphism in the included studies.

Author	Case		Control		*P*_HWE_	Association findings in case
	2G/1G	2G2G/2G1G/1G1G	2G/1G	2G2G/2G1G/1G1G		
Barlas	193/119	68/57/31	128/34	52/24/5	0.34	1G allele↑, 1G/1G, and 1G/2G genotype↑
Planello	134/96	45/44/26	118/116	26/66/25	0.16	1G allele↓, 1G/1G, and 1G/2G genotype↑
Abd-Allah	100/100	27/46/27	60/140	10/40/50	0.63	2G allele↑, 2G/2G, and 1G/2G genotype↑
Lepetsos	190/120	63/64/28	152/126	47/58/34	0.06	NS
Luo	223/189	66/91/49	151/219	29/93/63	0.58	NA
Yang	272/142	92/88/27	285/129	98/8920	0.97	NS
Geng	306/306	76/154/76	323/479	61/201/139	0.40	2Gallele↑, 2G/2G genotype↑

MMP: matrix metalloproteinase; HWE: Hardy-Weinberg equilibrium.

**Table 3:** Overall and subgroup meta-analysis of the association between MMP-1-1607 1G/2G (rs1799750) polymorphism and osteoarthritis risk under genetic models.

Subject	*N*	Allelic			Dominant			Recessive			Homozygote			Heterozygote		
		(2G vs. 1G)			(2G1G+2G2G vs. 1G1G)			(2G2G vs. 1G1G+1G2G)			(2G2G vs. 1G1G)			(2G1G vs. 1G1G)		
		OR (95% CI)	*P*	*P*_het_	OR (95% CI)	*P*	*P*_het_	OR (95% CI)	*P*	*P*_het_	OR (95% CI)	*P*	*P*_het_	OR (95% CI)	*P*	*P*_het_
*Total*		1.232 (0.889, 1.707)	0.21	<0.01	1.203 (0.801, 1.807)	0.373	0.001	1.478 (0.925, 2.6)	0.102	<0.01	1.512 (0.828, 2.761)	0.179	<0.01	1.088 (0.774, 1.529)	0.627	0.028
*Ethnicity*																
Caucasian	4	1.167 (0.627, 2.173)	0.626	<0.01	1.071 (0.483, 2.376)	0.866	0.001	1.408 (0.615, 3.224)	0.418	<0.01	1.342 (0.457, 3.945)	0.593	<0.01	0.985 (0.497, 1.951)	0.965	0.012
Asian	3	1.307 (0.899, 1.900)	0.161	0.002	1.314 (0.843, 2.048)	0.228	0.054	1.588 (0.872, 2.894)	0.131	0.002	1.703 (0.780, 3.716)	0.181	0.002	1.184 (0.850, 1.648)	0.317	0.224
*OA site*																
Keen OA	5	1.122 (0.716, 1.758)	0.615	<0.01	1.140 (0.637, 2.042)	0.659	<0.01	1.222 (0.695, 2.147)	0.486	<0.01	1.270 (0.549, 2.935)	0.576	<0.01	1.144 (0.734, 1.783)	0.552	0.031
TMJ OA	2	1.575 (1.259, 1.972)	<0.01	0.35	1.295 (0.741, 2.263)	0.363	0.139	2.411 (1.658, 3.504)	<0.01	0.757	2.313 (1.341, 3.991)	0.003	0.234	0.938 (0.487, 1.806)	0.847	0.106
*Total size*																
<300	4	1.167 (0.627, 2.173)	0.626	<0.01	1.071 (0.483, 2.376)	0.866	0.001	1.408 (0.615, 3.224)	<0.01	<0.01	1.342 (0.457, 3.945)	0.593	<0.01	0.985 (0.497, 1.951)	0.965	0.224
>300	3	1.307 (0.899, 1.900)	0.161	0.002	1.314 (0.843, 2.048)	0.228	0.054	1.588 (0.872, 2.894)	0.002	0.002	1.703 (0.780, 3.716)	0.181	0.002	1.184 (0.850, 1.648)	0.317	0.028
*Design*																
HCC	3	1.102 (0.453, 2.684)	0.83	<0.01	1.093 (0.358, 3.338)	0.876	<0.01	1.208 (0.420, 3.480)	0.726	<0.01	1.228 (0.251, 5.996)	0.8	<0.01	1.141 (0.499, 2.608)	0.755	0.024
PCC	4	1.323 (0.993, 1.761)	0.06	0.006	1.231 (0.842, 1.800)	0.284	0.06	1.713 (1.054, 2.783)	0.03	0.003	1.704 (0.943, 3.078)	0.077	0.007	1.035 (0.717, 1.494)	0.854	0.105
*Age*																
≥60	3	0.803 (0.457, 1.412)	0.45	<0.01	0.706 (0.295, 1.687)	0.433	0.009	0.815 (0.454, 1.464)	0.494	0.009	0.662 (0.234, 1.876)	0.438	0.003	0.799 (0.419, 1.524)	0.496	0.106
<60	4	1.635 (1.354, 1.974)	<0.01	0.201	1.622 (1.158, 2.271)	0.005	0.113	2.209 (1.718, 2.840)	<0.01	0.523	2.578 (1.798, 3.696)	<0.01	0.249	1.276 (0.868, 1.876)	0.216	0.077

MMP-1: matrix metalloproteinase-1; OA: osteoarthritis; TMJ: temporomandibular joint; HCC: hospital-based case-control study; PCC: population-based case-control study; *P*_het_: *P* value of heterogeneity.

## Abbreviations

MMP-1: Matrix metalloproteinase-1
OA: Osteoarthritis
TMJ: Temporomandibular joint
PCR-RFLP: Polymerase chain reaction-restriction fragment length polymorphism
OR: Odds ratio
CI: Confidence interval
HCC: Hospital-based case-control study
PCC: Population-based case-control study
HWE: Hardy-Weinberg equilibrium
ECM: Extracellular matrix.

## Supplementary Materials

Supplementary Materials Supplementary Table 1: Quality assessment of studies included in the meta-analysis using a Newcastle-Ottawa Scale. Supplementary Table 2: *P*-value of each subject for meta regression analysis in five genetic models.
