# Supplementary material for: Association between the MMP-1-1607 1G/2G Polymorphism and Osteoarthritis Risk: A Systematic Review and Meta-Analysis
Source: Biomed Res Int. 2020 May 20;2020:5190587. doi: 10.1155/2020/5190587 (PMC7273398; doi:10.1155/2020/5190587)
Supplement: Supplementary Materials — Supplementary Table 1: Quality assessment of studies included in the meta-analysis using a Newcastle-Ottawa Scale. Supplementary Table 2: P-value of each subject for meta regression analysis in five genetic models. [file 5190587.f1.pdf]

Supplementary table 1	Quality assessment of studies included in the meta-analysis using a Newcastle-Ottawa Scale							
	Author	Barlas 2009	Planello2011	Allah 2012	Lepetsos 2014	Luo 2015	Yang 2015	Geng 2018
Selection	Is the case definition adequate	1	1	1	1	1	1	1
	Representativeness of the cases	0	1	0	1	0	0	0
	Selection of Controls	0	1	0	0	1	1	1
	Definition of Controls	1	1	1	1	1	1	1
Comparability	Comparability of cases and controls	2	1	2	2	2	2	2
Exposure	Ascertainment of exposure	1	1	1	2	1	2	2
	Same method of ascertainment	1	1	1	1	1	1	1
	Non-Response rate	0	0	0	0	0	0	0
	Total	6	7	6	8	7	8	8

Supplementary table2 P-value of each subject for meta regression analysis in five genetic models

Subject	Allelic	Dominant	Recessive	Homozygote	Heterozygote
	2G vs 1G	2G1G+2G2G vs 1G1G	2G2G vs 1G1G+1G2G	2G2G vs 1G1G	2G1G vs 1G1G
Ethnicity（Caucasian, Asian）	0.732	0.807	0.675	0.741	0.816
OA site（keen OA,TMJ OA）	0.811	0.7	0.858	0.749	0.687
Method（PCR-RLFP,HRMA）	0.692	0.738	0.661	0.705	0.745
Design（HCC,PCC）	0.874	0.71	0.955	0.801	0.691
Sample（Blood, Buccal cell）	0.811	0.7	0.858	0.749	0.687
Surgery（Yes, No）	0.786	0.912	0.74	0.866	0.939
Total Size（<300,≥300）	0.732	0.807	0.675	0.741	0.816
Mean age（<60,≥60）	0.26	0.31	0.26	0.321	0.312
NOS (<7,≥7）	0.831	0.948	0.694	0.898	0.912

OA: osteoarthritis; TMJ: temporomandibular joint; PCR-RFLP: polymerase chain reaction-restriction fragment length polymorphism; HRMA: high resolution melting assay. HCC: Hospital based case–control study, PCC: Population based case–control study; NOS: Newcastle-Ottawa Scale
